# Mass Transfer Behavior of Benzene in Hierarchically Structured ZSM-5

**DOI:** 10.3389/fchem.2019.00502

**Published:** 2019-07-16

**Authors:** Xiuhong Meng, Chunhui Lin, Youhua Zhang, Huibo Qin, Shui Cao, Linhai Duan

**Affiliations:** Department of Chemical Engineering, Guangdong University of Petrochemical Technology, Maoming, China

**Keywords:** alkali treatment, hierarchical porous, ZSM-5, adsorption, diffusion

## Abstract

A series of ZSM-5 zeolites with hierarchical porous structure were synthesized using NaOH solutions treatment method. The structural and acidity properties of hierarchical ZSM-5 zeolites as-synthesized were characterized by X-ray diffraction (XRD), N_2_ adsorption, scanning electron microscope (SEM), NH_3_-temperature programmed desorption (TPD), and pyridine Fourier transform infrared spectroscopy (Py-FTIR). The adsorption and diffusion performances of benzene in hierarchical ZSM-5 zeolites were studied by an intelligent gravimetric analyzer (IGA). It was found that mass transfer (adsorption and diffusion) performance of benzene was significantly affected by synergetic effect of hierarchical structure, acid amount, acidity, adsorption sites of ZSM-5 zeolites. After suitable alkali treatment, the crystal structure of ZSM-5 was retained and finely tailored. Hierarchical ZSM-5 was obtained with a uniform size of mesoporous and microporous structure. Acidity of hierarchical ZSM-5 zeolites was improved, which produced more adsorption sites and thus increased the adsorption performance of benzene in hierarchical ZSM-5. As a result, connectivity in hierarchical ZSM-5 was improved with increasing of mesopores in hierarchical ZSM-5. Hierarchical ZSM-5 well-contributed to the adsorption performance of benzene on active sites and improved catalytic performance of hierarchical ZSM-5.

## Introduction

ZSM-5 are commonly used in industry due to the thermal stability and high surface area, appropriate pore size. ZSM-5 possesses unusual hydrophobicity, leading to potential applications in the separation of hydrocarbons from polar compounds, such as water and alcohols. These unique properties are responsible for their application as catalysts in many major chemical processes (Tang et al., 2006; Duan et al., 2013; Mu et al., 2013; Nandan et al., 2014; Hasan et al., 2015). The relatively narrow channels in ZSM-5, however, may lead to diffusion limitations for large reactant molecules. Several methods, such as synthesizing new zeolites with larger pores (Mu et al., 2013), synthesizing meso-microporous or micro-microporous composite zeolites (Corma, 2003; Christensen et al., 2007; Zhou et al., 2017), preparing smaller zeolite particles (Mitchell and Pérez-Ramírez, 2011), and modifying the pore structure of zeolites (Fernandez et al., 2010; Duan et al., 2012), have been proposed to overcome inherent diffusion limitations. Among these methods, desilication by innovative post-treatment method, has been extensively considered because that can enlarge the micropores of ZSM-5 slightly and shorten the diffusion path length of molecules in the micropores (Groen et al., 2006; Zhao et al., 2008; Chal et al., 2011; Lopez-Orozco et al., 2011; Sadowska et al., 2012; Mintova et al., 2013; Ma et al., 2019). This facilitates guest molecule to diffuse out of the ZSM-5 and form final liquid products rather than being adsorbed in the pores to form coke. As a consequence, it is highly needed to develop new materials with hierarchical architecture of porosity (van Donk et al., 2003; Ma et al., 2019). The large external surface provides more active sites, and moreover offers a short diffusion path for guest molecules (Mintova et al., 2013).

In order to improve the catalyst efficiency in chemical reactions, desilication by alkali-treatment was found to be a very effective and reproducible method for preparation of mesoporous zeolites without significant damages both in crystallinity and acidity (Groen et al., 2006; Chal et al., 2011; Lopez-Orozco et al., 2011). Previous studies showed that desilication of ZSM-5 can improve the accessibility of acid sites in zeolites, and results in redistribution of the framework Al atoms (Sadowska et al., 2012). Meanwhile, the diffusion capability of large molecules in zeolites channels can also be improved by the introduction of mesoporosity, which has been indirectly proven by enhanced catalytic performance. Zhao et al. (2008) reported that both the adsorption amount and the adsorption rate increased outstandingly in the mesopore structured ZSM-5 generated by alkali-treatment compared with the parent ZSM-5. It was found that conversion of cumene in mesopore ZSM-5 catalyst has increased a lot compared with the conventional ZSM-5 catalysts. These findings indicate that the catalytic selectivity, conversion rate are very relevant to the diffusion behavior in the zeolite. However, the relationship between the diffusion mechanism and adsorption sites is not well-understood. Ogura et al. (2001) proposed that the treated ZSM-5 in NaOH alkali solution can change the structure and acidic properties of ZSM-5. The mesopores with a uniform size can be formed on the zeolite, while the microporous structure remained. The acidic property has very little change quantitatively or qualitatively, even though the catalytic activity for cracking of cumene was enhanced by the alkali-treatment due to that the adsorptive and diffusive performance of cumene are increased by the creation of mesopores in microporous ZSM-5.

Benzene, which can be synthesized to a series of derivatives of benzene, is very important to make chemical raw materials. Benzene is an important product in petrochemical industry for increasing aromatics production and octane number of gasoline. In this study, benzene was selected to study the mass transfer performance in hierarchically structured ZSM-5. However, the diffusion mechanism of aromatic hydrocarbons that result in increased catalytic conversion is not well-understood. Furthermore, the adsorption dynamic and thermodynamic such as the activation energy and adsorption heat, which are helpful in understanding the diffusion process and the catalytic performance, are not available for hierarchically ZSM-5. Our group has studied the adsorption property of several samples in zeolites and corresponding research (Meng et al., 2009; Sui et al., 2013; Qin et al., 2014a,b).

Hence, in this contribution we aims to reveal the intrinsic correlation between the mass transfer performance of benzene on hierarchical ZSM-5 and adsorption sites, as well as adsorption sites and acidic characteristics by using a high precision intelligent gravimetric analyzer (IGA), pyridine adsorption Fourier-transform infrared spectroscopy (pyridine-IR) with hierarchical ZSM-5 as adsorbent and benzene as adsorbate.

## Experiment

### Preparation of Mesoporous Zeolites

Commercially available NaZSM-5 zeolite was used as parent material prepared by hydrothermal synthesis method. HZSM-5 prepared by ion exchange with (NH_4_)_2_SO_4_ aqueous solution was designated as“B0” in this work. Three groups were chosen as representative samples by orthogonal experiment design method. Group I was treated with 0.2 mol/L NaOH at 343 K for 3 h. Group II was treated with 0.25 mol/L NaOH at 343 K for 4 h. Group III was treated with 0.4 mol/L NaOH at 363 K for 5 h. Firstly, conventional ZSM-5 was mixed with a series of NaOH aqueous solutions. After a certain period of alkali treatment, the slurry was cooled rapidly to room temperature in an ice bath, filtered and washed with distilled water until a neutral pH of filtrate was obtained. The remaining solid was dried in 393 K for 10 h.

Alkali-treated NaZSM-5 was exchanged with (NH_4_)_2_SO_4_ aqueous solution, followed by filtering and rinsing with distilled water to remove sodium ions. This procedure was repeated twice to obtain NH_4_ZSM-5. After drying at 393 K for 10 h, NH_4_ZSM-5 was calcined in static air at 823 K for 5 h to form the HZSM-5. These (NH_4_)_2_SO_4_ treatment samples obtained at different conditions were designated by B1 [temperature 343 K, time 3 h, (NH_4_)_2_SO_4_ concentration 0.2M], B2 [temperature 343 K, time 4 h, (NH_4_)_2_SO_4_ concentration 0.25M], B3 [temperature 363 K, time 5 h, (NH_4_)_2_SO_4_ concentration 0.4M], respectively.

### Characterization

The crystallinity of parent ZSM-5 and hierarchically ZSM-5 was characterized by X-ray diffraction. XRD measurements were taken in a XRD-6000 using Cu κα radiation at 100 mA and 30 mV. N_2_ adsorption/desorption isotherms were recorded on a Quanta chrome Autosorb Automated Gas Sorption analyzer ASAP2020 at 77 K. The mesoporous volume and specific surface were evaluated by the t-plot method. The total surface area was calculated according to BET isotherms.

Temperature-programmed NH_3_ desorption (NH_3_-TPD) was measured by an automatic chemical adsorption instrument, aiming to investigate acidity distribution. The nature of acid sites was investigated using pyridine as the probe molecule. The FTIR spectra of pyridine adsorption were recorded using an FTIR spectrometer.

## Results and Discussion

### Textural and Features of the Alkali-Treated ZSM-5

**Figure 1:**
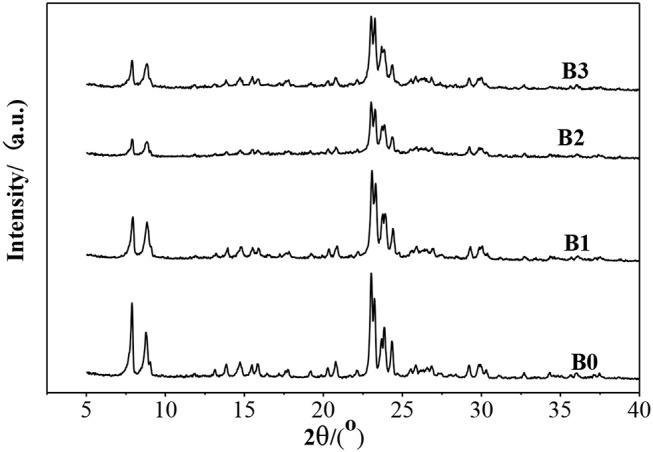
XRD patterns of ZSM-5 and alkali-treatment zeolites.

XRD (Figure 1) showed that the ZSM-5 structure was well-retained after alkali-treatment, but the intensity of most peaks decrease slightly, indicating that alkali-treatment did not destroy the crystal structure of ZSM-5, but the structure was finely modulated.

**Figure 2:**
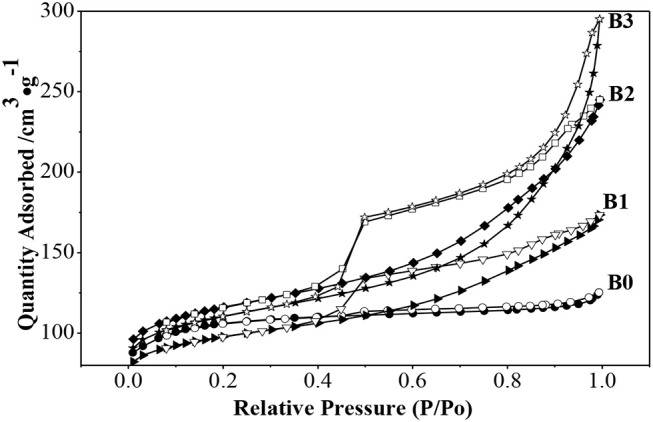
N_2_ adsorption and desorption isotherms of alkali-treatment ZSM-5 at −196°C.

**Figure 3:**
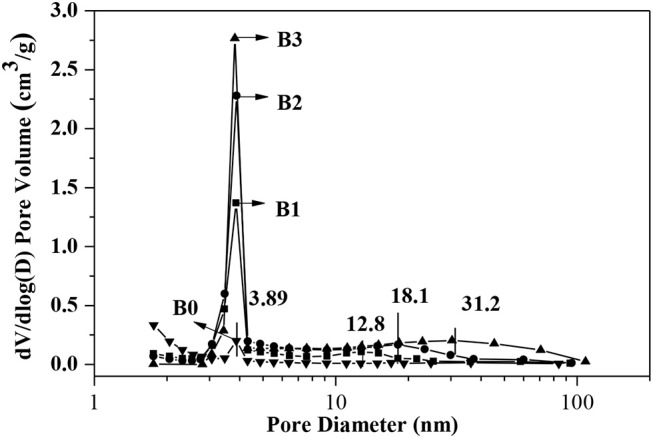
Pore size distributions of alkali-treatment ZSM-5 calculated by BJH method.

N_2_ adsorption isotherms and pores-size distribution curves of the samples are shown in Figures 2, 3 respectively. The hysteresis loop of the isotherm changed with different alkali-treatment conditions. N_2_ isotherms (Figure 2) clearly show that all the isotherms exhibit type IV, which are the typical characteristics of mesoporous materials (Verboekend and Pérez-Ramírez, 2011). The BET surface area data as well as the micro-and mesoporous volume determined by a t-plot method were shown in Table 1. Remarkably, the specific surface area of the alkali-treatment ZSM-5 showed an increasing trend and the mesoporous volume increased from 0.08 to 0.35 cm^3^/g. This increase is due to the creation of the mesoporous with a slight decrease in the microspore volume (0.114 vs. 0.106 cm^3^/g for fresh H-ZSM-5 and hierarchically H-ZSM-5, respectively). The decrease in micropore volume was caused by desilication of ZSM-5 and thus induced the minor amorphization in the process of alkali-treatment (Verboekend et al., 2011). XRD patterns results showed the hierarchically structured ZSM-5 can be obtained by alkali-treatment methods while preserving the MFI micropore structure.

**Table 1:** Chemical and physical properties of hierarchically ZSM-5.

**Sample**	**D_**aver**_**	**S_**BET**_**	**S_**mic**_**	**S_**Ext**_**	**V_**P**_**	**V_**meso**_**
	**nm**	**m^**2**^/g**	**m^**2**^/g**	**m^**2**^/g**	**cm^**3**^/g**	**cm^**3**^/g**
B0	2.85	327	212	115	0.194	0.080
B1	4.59	306	189	118	0.268	0.168
B2	5.27	367	212	155	0.379	0.267
B3	6.62	350	201	149	0.456	0.350

**Figure 4:**
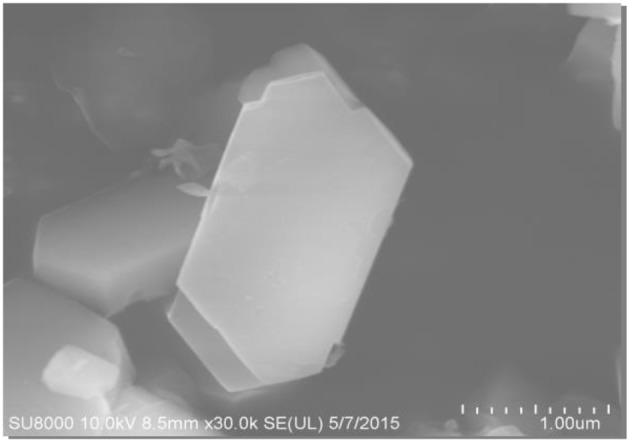
SEM of fresh ZSM-5.

**Figure 5:**
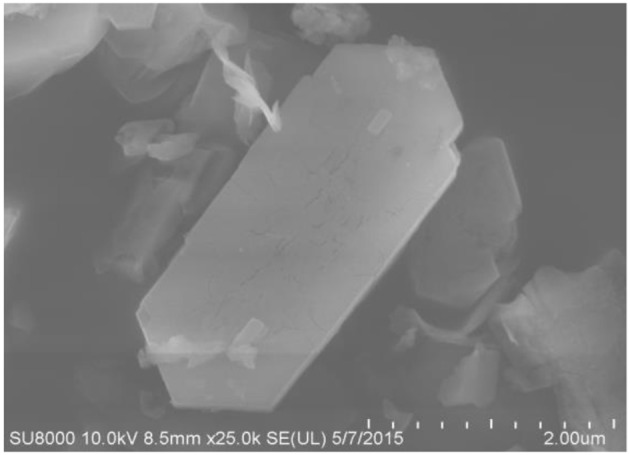
SEM of alkali-treatment ZSM-5.

**Figure 6:**
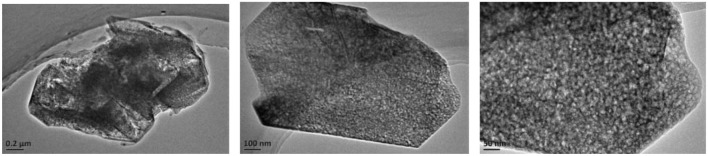
TEM of alkali-treatment ZSM-5.

The SEM and TEM images of these samples are shown in Figures 4–6, respectively. The morphology of ZSM-5 is rectangular or hexagonal (Figure 4). With increase of the concentration of NaOH, ZSM-5 can still maintain its crystal structure, which is in accordance with the XRD results. The magnified SEM image shows that it has a mesoporous-like morphology, which is in accordance with the N_2_ adsorption-desorption results.

### Effect of Alkali Treatment on Acidic Properties of ZSM-5

**Figure 7:**
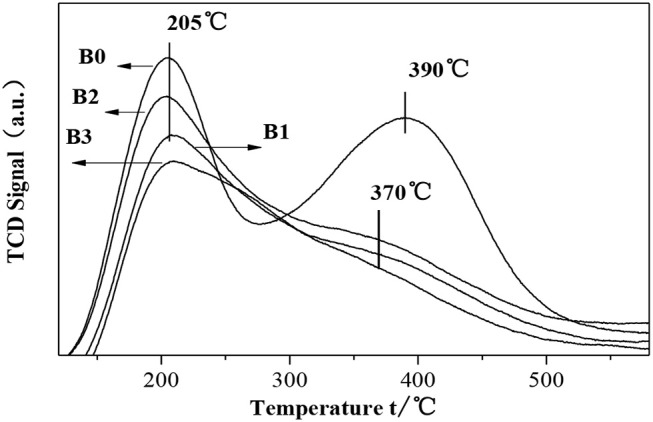
NH_3_-TPD spectra of parent ZSM-5 (B0) and hierarchically ZSM-5 (B1, B2, B3).

The NH_3_-TPD results of fresh HZSM-5 and hierarchically HZSM-5 are shown in Figure 7. We can see that the desorption profiles of parent H-ZSM-5 shows two maxima, which are at 207 and 390°C, respectively. The first maxima belongs to the weakly absorbed NH_3_ molecules, whereas the second one originates from B acid sites. In case of hierarchically HZSM-5, the strong acid sites are more pronounced than that in parent H-ZSM-5.

**Figure 8:**
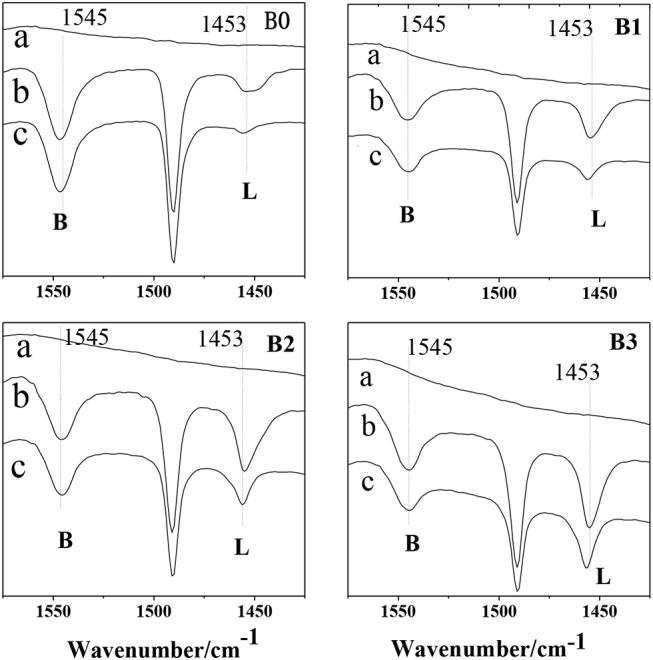
Py-FTIR spectra of parent ZSM-5 (B0), hierarchically ZSM-5 (B1, B2, B3) at different temperatures. (a): background; (b): 150°C; (c): 400°C.

Figure 8 show the FITR spectra of HZSM-5 samples after desorption at difference temperatures. The adsorption at 1,545 and 1,453 cm^−1^ are assigned to the B and L acid sites, respectively. Apparently, a large number of L acid sites are formed in the zeolites after desilication. The results reveals that the weak acidity observed in NH_3_-TPD (Figure 7) has a clear Lewis nature and can be ascribed to Al in extraframework of hierarchically ZSM-5, which is produced during desilication. In summary, it can safely say that the higher the alkali concentration is, the stronger the Lewis acidity. For the hierarchically HZSM-5 (B1, B2, B3), the acidity of B2 sample is the largest, which can provide more adsorption sites for molecular diffusion in hierarchically ZSM-5.

### Adsorption, Diffusion of Benzene on Hierarchically Structured ZSM-5

**Figure 9:**
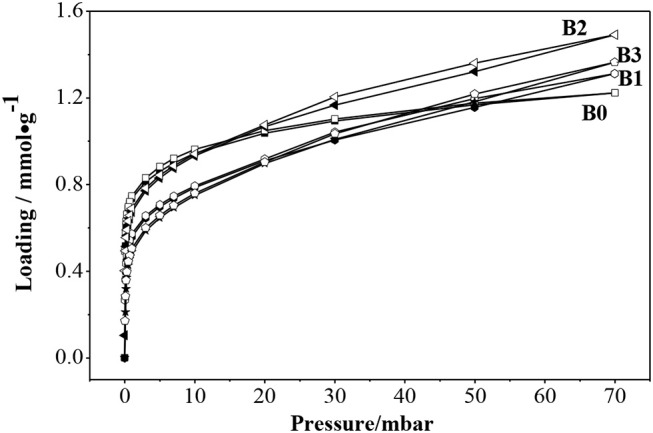
Adsorption isotherms of benzene on fresh ZSM-5 and hierarchically ZSM-5 at 30°C.

Figure 9 showed the adsorption isotherms of benzene at 30°C in ZSM-5 (B0) and hierarchically ZSM-5 (B1, B2, B3). The isotherm of ZSM-5 showed typical Langmuir Type-I adsorption, which reached saturation at low pressure and increased slightly with further increase of pressure. The isotherms in hierarchically ZSM-5 (B1, B2, B3) showed hysteresis loops which are different from the isotherms in B0. It can be seen that the adsorption amount of benzene in B2 is higher than that in B1 and B3, the reason may be that the specific surface area of micro-mesoporous in B2 is higher than that in B1 and B3. The adsorption amount of benzene in B2 continued to increase with the increasing pressure. The obvious hysteresis loop in the adsorption and desorption isotherm was mainly caused by the capillary condensation of porous adsorbent.

It can be seen that the adsorption sites is increased in hierarchically ZSM-5 according to the benzene adsorption amount. The highest value of S_meso_, as well as the highest total volume was obtained for B2. Benzene molecule prefers to adsorbe on the effective adsorption sites, which in accordance with the largest acid amount of B2. Although B3 has a broader mesoporous distribution, it initially promotes benzene molecules to enter its pore channels. When enough benzene molecules are adsorbed in the effective adsorption sites, the hierarchically ZSM-5 cannot accommodate more molecules due to the interaction forces between molecules and pore walls. So the adsorption performance of benzene on hierarchical ZSM-5 was correlated not only with the acidity of ZSM-5, but also with hierarchical structure.

**Figure 10:**
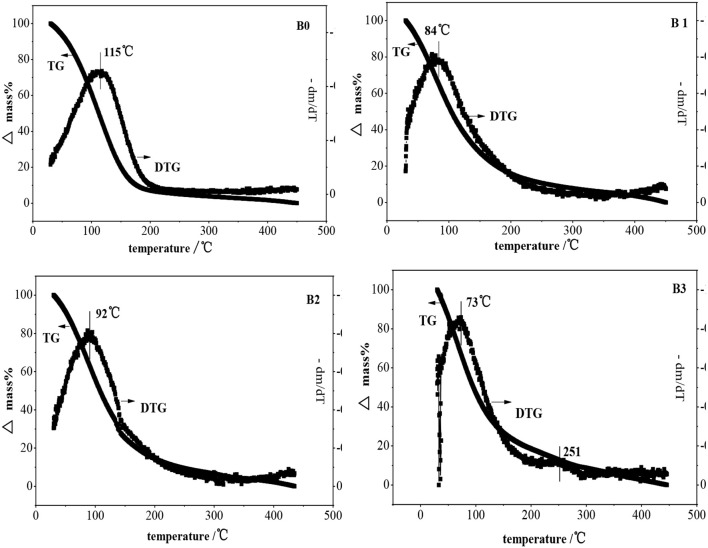
Temperature programmed desorption of benzene in ZSM-5 and hierarchically ZSM-5.

The temperature programmed desorption of benzene in ZSM-5 and hierarchically ZSM-5 is shown in Figure 10. It can be seen that only one desorption peak is showed in the Derivative Thermogravimetry (DTG) curve of benzene in ZSM-5, suggesting that there is only one interaction mode between benzene and ZSM-5 before and after alkali-treatment. Benzene can be completely desorbed before 200°C, indicating that the adsorption interaction belongs to physical adsorption (the van der Waals force) between benzene molecule and ZSM-5, which is relatively weak interaction. Compared with the hierarchically ZSM-5 zeolite, the parent ZSM-5 has stronger acidic center and larger acidity amount, i.e., more adsorption sites and larger interaction force, so the desorption temperature is relatively high. Moreover, the adsorption diffusion rate in parent ZSM-5 is the smallest. Because there is no much mesoporous and catalytic adsorption sites in it, the diffusion path is easily blocked.

**Figure 11:**
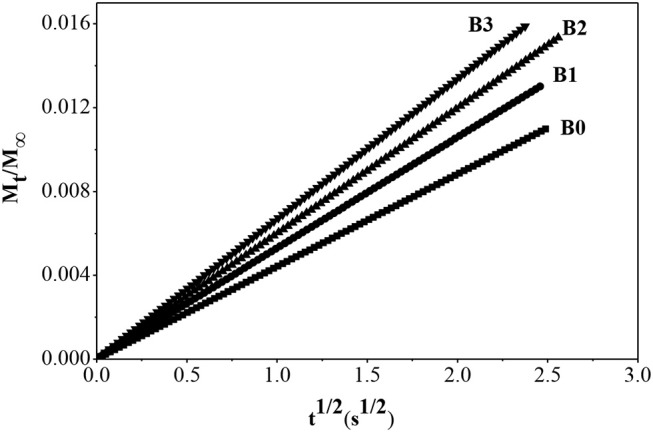
Adsorption rate curve of benzene molecule in fresh ZSM-5 and hierarchically ZSM-5 at 30°C.

With the increase of alkali concentration for preparing hierarchically ZSM-5, partial Al atoms can be removed from the framework of ZSM-5, which is adsorbed on the surface of hierarchically ZSM-5, and thus provided more L acid sites. Meanwhile, the mesopores and pore volume increased gradually, which provided more effective adsorption sites for benzene molecules and shortened the diffusion path. The adsorption kinetics of benzene molecule in fresh ZSM-5 and hierarchically ZSM-5 at 30°C are showed in Figure 11, and the diffusion time constants is showed in Table 2. It can be seen that the diffusion rate of benzene in hierarchically ZSM-5 increased significantly. B3 has more mesopores and pore volume in these three kinds of hierarchically ZSM-5 as synthesized, which provides a fast adsorption and diffusion path for benzene diffusion. And thus, benzene has the largest adsorption capacity and the fastest diffusion rate in B3. While, the TG-DTG curve shows that the interaction force between benzene molecule and B3 is the smallest, suggesting that the adsorption and diffusion performance of benzene is not only depended on the structure of hierarchically ZSM-5 but also related to the effective adsorption sites in its channels. The TG-DTG curves of B2 show that the desorption temperature of B2 is highest in these three kinds of hierarchically ZSM-5, implying that the interaction force is greatest in B2. The percentages of mesopores in B2 is lower than B3, and so its micro-mesoporous connectivity is poorer than B3, so the diffusion time constant of benzene in B2 is smaller than that in B3. These results also imply that adsorption and diffusion performance of benzene is not only related to the connectivity of channels in hierarchically ZSM-5, but also depend on effective adsorption sites in it.

**Table 2:** Diffusion time constants of benzene on ZSM-5 zeolites and hierarchically ZSM-5 at 30°C.

**D/r^2^(S^−1^)**				
Sample	B0	B1	B2	B3
Benzene	4.21 × 10^−3^	6.05 × 10^−3^	7.76 × 10^−3^	9.68 × 10^−3^

## Conclusion

The hierarchically ZSM-5 with good connectivity was successfully synthesized by alkali treatment method. With the increase of alkali concentration, the mesoporous volume and surface area in hierarchically ZSM-5 gradually enhanced, and the adsorption surface area correspondingly increased, which reduced the diffusion resistance of molecules in the pore channels of hierarchically ZSM-5. By investigating the adsorption and diffusion properties of benzene in hierarchically ZSM-5, we can safely conclude that the adsorption capacity and diffusion rate in hierarchically ZSM-5 are not only related to the hierarchically pore structure, but also depended on the effective adsorption sites. Hierarchically ZSM-5 with high acidity has more effective adsorption sites and better performances, which are conducive to the adsorption and diffusion performances of benzene molecules. The good connectivity in hierarchical ZSM-5 improved the mass transfer performances of benzene, which was beneficial to the catalytic performance of ZSM-5 catalyst efficiently.

## Data Availability

All datasets generated for this study are included in the manuscript/supplementary files.

## Author Contributions

All authors listed have made a substantial, direct and intellectual contribution to the work, and approved it for publication.

### Conflict of Interest Statement

The authors declare that the research was conducted in the absence of any commercial or financial relationships that could be construed as a potential conflict of interest.
